# The neural correlates of topographical disorientation—a lesion analysis study

**DOI:** 10.1002/acn3.51967

**Published:** 2024-01-17

**Authors:** Eva Blondiaux, Andreas Diamantaras, Rahel Schumacher, Olaf Blanke, René Müri, Lukas Heydrich

**Affiliations:** ^1^ Laboratory of Cognitive Neuroscience Brain‐Mind Institute, School of Life Sciences, Ecole Polytechnique Fédérale de Lausanne Lausanne Switzerland; ^2^ Center for Neuroprosthetics School of Life Sciences, Ecole Polytechnique Fédérale de Lausanne Lausanne Switzerland; ^3^ Department of Neurology Inselspital, Bern University Hospital, University of Bern Bern Switzerland; ^4^ CORE Lab, Psychosomatic Competence Center, Department of Neurology Inselspital. Bern University Hospital, University of Bern Bern Switzerland; ^5^ Department of Neurology Inselspital, University Neurorehabilitation, Bern University Hospital, University of Bern Bern Switzerland; ^6^ Department of Neurology University Hospital Geneva Geneva Switzerland

## Abstract

Topographical disorientation refers to the selective inability to orient oneself in familiar surroundings. However, to date its neural correlates remain poorly understood. Here we use quantitative lesion analysis and a lesion network mapping approach in order to investigate seven patients with topographical disorientation. Our findings link not only the posterior parahippocampal gyrus (PHG) and retrosplenial cortex but also the lingual gyrus, the precuneus and the fusiform gyrus to topographical disorientation. We propose that topographical disorientation is due to the inability to integrate familiar landmarks within a framework of allocentric and egocentric orientation, supported by a neural network including the posterior PHG, the retrosplenial and the lingual cortex.

## Introduction

Topographical disorientation, also referred to as topographagnosia, heading disorientation or topographical amnesia, is a rare neurological condition characterized by selective topographical disorientation, which entails the inability to orient oneself in familiar surroundings.^1^ While most authors have suggested that topographical disorientation is due to a failure of allocentric orientation (i.e. orientation relative to external reference points),^2^, ^3^ others have demonstrated that egocentric orientation (i.e. orientation relative to the body) can also be affected.

Topographical disorientation has been reported after cerebrovascular stroke,^4^ traumatic brain injury^5^ and in dementia.^6^ However, these reports were in the majority single case studies^4^ or small case series,^7^, ^8^ highlighting lesions of the temporo‐occipital regions,^8^ particularly the parahippocampal gyrus (PHG),^2^, ^3^, ^4^ the cingulate cortex^9^ and the lingual gyrus.^7^ Although single case studies allow in‐depth evaluation of patients with topographical disorientation (i.e. the demonstration of impaired egocentric orientation by Nyffeler et al. using an oculomotor task^4^), the neural correlates of topographical disorientation remain poorly understood, given the lack of studies employing quantitative lesion analysis in a larger patient sample. Also, to date no study has investigated the underlying neural networks of topographical disorientation.

Therefore, in this study, we systematically examined a group of seven patients with structural brain damage and topographical disorientation using quantitative lesion analysis^10^ and a lesion network mapping approach.^11^ We aimed to better characterize and understand the neural correlates, specifically the neural network underlying topographical disorientation.

## Materials and Methods

### Patient characteristics and clinical workup

We selected patients presenting with topographical disorientation, from a population of patients suffering from structural brain damage treated at the Neurorehabilitation Unit of the University Hospital of Bern between 2005 and 2018. As a control group, patients with general spatial disorientation without signs of topographical disorientation were selected. Patients with dementia and psychiatric illness were excluded from the analysis. Inclusion and exclusion criteria were established prior to data analysis. Ethical approval was obtained from the Ethical committee of the University Hospital of Bern. The study procedures were not pre‐registered prior to the research being conducted.

Patients' demographic information characteristics (age, sex, handedness and psychiatric comorbidities) and the results of neuropsychological evaluation (executive functions, verbal and non‐verbal memory, attention and visuo‐constructive functions) were compared between the two groups. We used a *χ* ^2^ test for independent samples or the Fisher's exact test if the total number of expected observations in the contingency table was less than 20, all expected cell frequencies were less than five, or any expected cell had fewer than one observation. *P*‐values were corrected for multiple comparisons using the Bonferroni correction.

### Lesion overlap

In order to determine the maximal zone of lesion overlap, a lesion mapping approach was applied in each of the patients as described previously.^10^

Anatomical structures were labelled according to the Automated Anatomical Labelling (AAL) atlas implemented in MRIcron^12^ and compared using a *χ* ^2^ test, or the Fisher's exact test, if the expected contingency table frequencies were low.

In order to illustrate the neuroanatomical correlates underlying topographical disorientation, we subsequently traced the lesion volume for each patient on the T1 template using MRIcron. This allowed us to perform a simple voxel‐based lesion overlap analysis establishing the anatomical subregions of maximal overlap for each group. No statistical lesion overlap comparison (e.g. voxel‐based lesion symptom mapping) was performed given the low number of patients in each group. For further details, please refer Data S1.

### Lesion network mapping

In order to further investigate the network associated with topographical disorientation, lesion network mapping analysis was applied.^11^ In short, this method uses publicly available normative resting state data from healthy subjects (*N* = 126)^13^ to investigate the functional connectivity of the lesion locations causing a symptom (here, topographical disorientation and spatial disorientation (control group).^11^ This enables to identify the network of connected brain regions associated with the symptom. The lesions were used as seed region of interest in the resting state analysis, and the resulting maps were thresholded with T > ±4.5 (*P* < 0.00005 uncorrected) as used previously,^11^ binarized and overlapped to determine the brain regions functionally connected to all lesions. This analysis was applied to both the groups of patients.

To assess whether the brain regions were specific to topographical disorientation, lesion maps from the two groups (topographical disorientation and the control group) were compared using the Liebermeister test, using voxel‐based lesion mapping (VLSM).^12^ The specificity analysis was restricted to the topographical disorientation lesion derived network (i.e. brain regions connected to all lesion locations). Permutation (*N* = 4000)‐derived FWE correction was used to determine significant voxels (using voxels damaged in at least 30% of the lesions).

## Results

### Demographics and clinical characteristics

Seven patients fulfilled the criteria for topographical disorientation. All patients showed a marked inability to navigate in a familiar (at home, neighbourhood) or more or less familiar (e.g. hospital) environment while general spatial orientation was preserved. As a control group, seven patients with general spatial disorientation without signs of topographical disorientation were selected. For further details, refer to Table 1.

**Table 1:** Demographic characteristics and clinical details.

	Topographical disorientation (*n* = 7)	Control group (*n* = 7)	*P*‐value
Demographics			
Gender (male/female)	7/0	6/1	n.s.
Age at evaluation (mean years ± SD)	61.7 ± 4.9	66.3 ± 17.3	n.s.
Type of lesion (*n*)			n.s.
Ischemic	5	3	n.s.
Traumatic brain injury/ruptured Aneurysm	1	3	n.s.
Malignancy	0	1	n.s.
Encephalitis	1	0	n.s.
Location of the lesion (*n*)			
Right/left/both hemisphere(s)	5/0/2	6/0/1	
Cognitive deficits in neuropsychological evaluation (*n*)	(*n* total = 6)	(*n* total = 6)	
Executive functions	4	6	n.s.
Memory (verbal)	4	6	n.s.
Memory (non verbal)	6	4	n.s.
Attention	5	4	n.s.
Visual perception and construction	6	4	n.s.
Neurological examination			
Aphasia	1	0	n.s.
Neglect	2	2	n.s.
Hemianopsia	5	2	n.s.
Sensorimotor hemi‐syndrome	4	3	n.s.

### Neuropsychological testing

Neuropsychological testing was available in 12 patients (six patients with topographical disorientation and six patients from the control group). None of the neuropsychological tests showed a significant difference between the two patient groups (see Table 1 and Table S1).

### Lesion overlap

**Figure 1:**
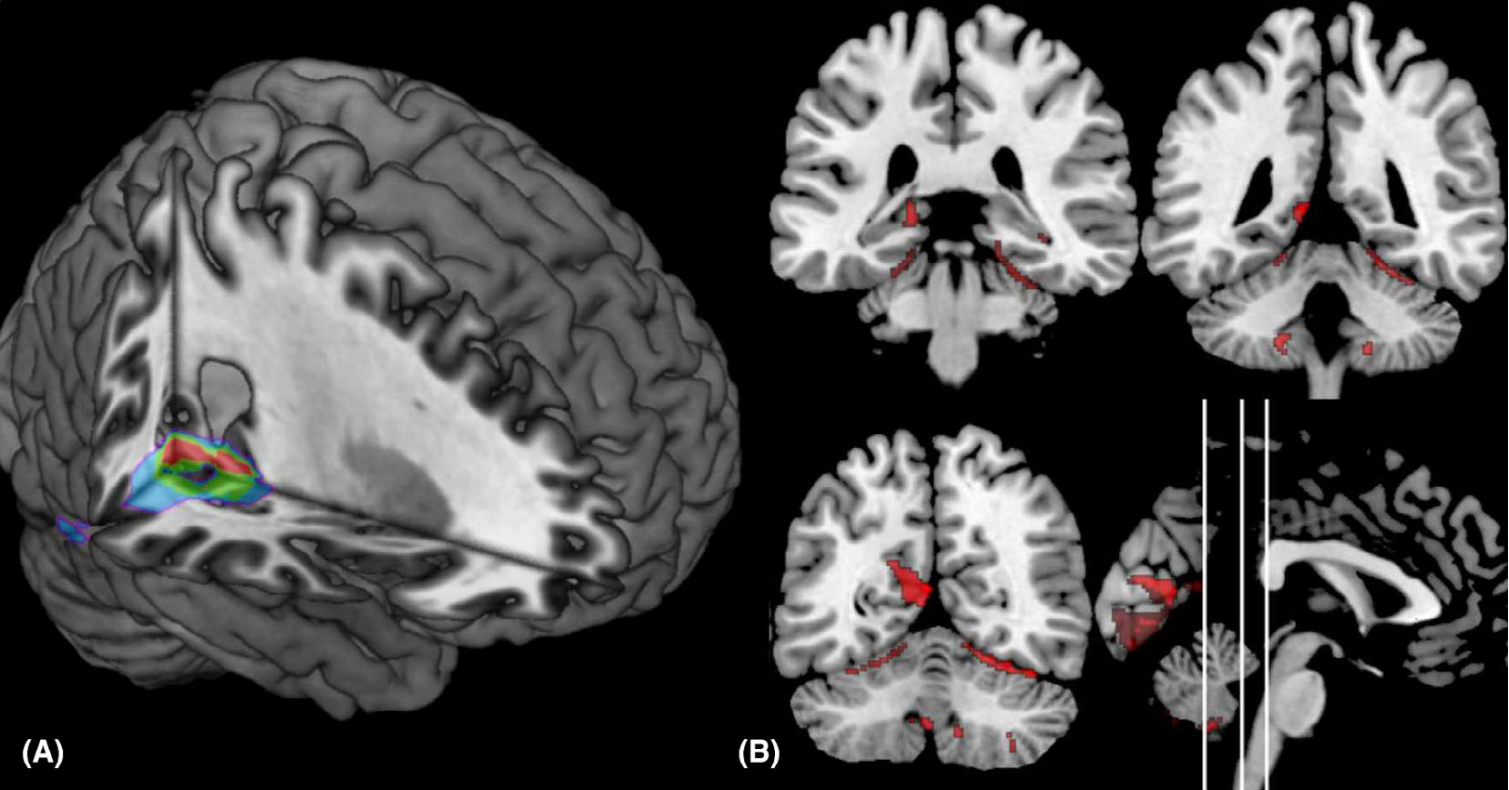
(A) Maximal overlap. (B) Brain regions connected specifically to all lesions causing topographical disorientation. The coronal slices correspond to: *Y* = −34, *Y* = −44 and *Y* = −58.

Patients presenting with topographical disorientation as well as the patients from the control group all suffered from a lesion in the right hemisphere. Figure 1 shows the results of the voxel‐based overlap analysis.

In patients with topographical disorientation, the right medial temporal lobe, notably the posterior PHG (Brodmann area 27), but also the lingual gyrus (Brodmann area 19), the retrosplenial cortex (Brodmann area 30) and the fusiform gyrus (Brodmann area 37) are maximally implicated in six of seven patients (centred at MNI *x* = 25, *y* = −41, *z* = −4). In the control group, the right medial temporal lobe, notably the right hippocampus, but also the right temporal pole and the right prefrontal cortex were affected (MNI *x* = −24, *y* = −5, *z* = −26).

### Lesion network mapping

Brain regions functionally connected to all lesion locations causing topographical disorientation included the bilateral lingual cortex/precuneus, the cerebellum, the bilateral hippocampus, the left fusiform gyrus and the right thalamus (Figure 1B and Table S2). These regions were specifically associated with lesions causing topographical disorientation compared to the lesion causing spatial disorientation without signs of topographical disorientation.

All data are available under https://osf.io/5qxft/?view_only=a1bd1b16ee044990a17978acf93bd3ba.

## Discussion

Here we systematically studied the clinical, neuropsychological and neuroanatomical correlates in patients with topographical disorientation. All patients showed a marked inability to navigate in a familiar environment while general spatial orientation was preserved. Visuo‐spatial disorders including visual field deficits and neglect were both frequent in patients with topographical disorientation and patients from the control group with general spatial disorientation. Neuropsychological testing revealed no significant difference in cognitive abilities between the two groups. Our lesion analysis and lesion network mapping highlight the role of the right medial temporal lobe, notably the posterior PHG and retrosplenial cortex, but also the lingual gyrus, the precuneus and the fusiform gyrus in topographical disorientation.

The right PHG has been repeatedly linked to topographical disorientation.^14^, ^15^ It has been suggested that the PHG plays an important role in egocentric and allocentric orientation. For example, patients with right‐sided posterior PHG lesions are impaired on a task involving allocentric orientation, for example, a virtual maze acquisition task.^16^ Others have found that lesions of the PHG affect an oculomotor task requiring egocentric orientation.^17^ Our findings are in line with these previous studies.

Lesion analysis also highlighted the retrosplenial cortex. The retrosplenial cortex is believed to contain not only neurons such as head direction cells, but also cells supporting spatial and contextual encoding, thus playing an important role in the processing and integration of egocentric and allocentric information and goal‐directed navigation.^18^

Crucially, to locate oneself within the environment requires the processing and integration of both egocentric and allocentric spatial information.^19^ It has been suggested that the combination of impaired egocentric and allocentric mapping of the environment results in topographical disorientation.^3^, ^4^, ^14^

Our lesion network analysis suggested a functional connectivity of all lesion locations causing topographical disorientation, mainly with the bilateral lingual cortex. The lingual cortex has been linked to landmark representation and the recognition of familiar objects.^7^ Given that the representation of landmarks and objects is important in order to navigate in a familiar environment, we argue that topographical disorientation (a) results from the inability to integrate familiar landmarks within the framework of allocentric and egocentric orientation and (b) is likely caused by disconnection of egocentric and allocentric information due to lesions of a neural network including the PHG, the retrosplenial cortex and the lingual cortex.

It is important to acknowledge the limitations of our study. The relatively small number of patients is due to the rarity of topographical disorientation, potentially limiting the generalizability of our findings. Moreover, a prospective study design would allow for a more systematic assessment, including comprehensive neuropsychological evaluations and standardized imaging protocols. This could provide a better understanding of other factors contributing to topographical disorientation, as many patients with similar lesion patterns do not exhibit such symptoms.

In conclusion, our study suggests that topographical disorientation arises from a combination of lesions in the medial temporal lobe, disrupting the integration of egocentric and allocentric information within a neural network including the PHG, the retrosplenial cortex and the lingual cortex. Further research with a larger sample size is warranted to validate and expand upon these results.

## Author contributions

**Eva Blondiaux**—Formal analysis, Investigation, Data Curation, Writing Original draft, Writing Review and Editing. **Andreas Diamantaras**—Formal analysis, Software, Data Curation, Writing Original draft. **Rahel Schumacher**—Formal analysis, Investigation, Writing Original draft. **Olaf Blanke**—Conceptualization, Investigation, Writing Original draft, Writing Review and Editing. **René Müri**—Writing Original draft, Project administration. **Lukas Heydrich**—Conceptualization, Methodology, Formal analysis, Investigation, Writing Original draft, Writing Review and Editing, Supervision, Project administration, Funding acquisition.

## Conflict of Interest

The authors report no conflicts of interest and no financial relationships relevant to the manuscript.

## Supporting information


**Data S1.** Supporting information.

**Table S1.** Neuropsychological profile of patients with TD and the control group.

**Table S2.** Brain regions functionally connected to all lesion locations in TD.
