# Supplementary material for: The neural correlates of topographical disorientation—a lesion analysis study
Source: Ann Clin Transl Neurol. 2024 Jan 17;11(2):520–4. doi: 10.1002/acn3.51967 (PMC10863913; doi:10.1002/acn3.51967)
Supplement: Supplementary file 1 — Data S1. Supporting information. [file ACN3-11-520-s002.docx]

Supplementary online material for

**The neural correlates of topographical disorientation – a lesion analysis study**

Blondiaux, E^1,2*^, Diamantaras, A^3,4*^, Schumacher, R^6,^ Blanke, O^1,2,5^, Müri, R^3, 6^, Heydrich, L ^3,4^

^1^ Laboratory of Cognitive Neuroscience, Brain-Mind Institute, School of Life Sciences, Ecole Polytechnique Fédérale de Lausanne, Switzerland

^2^ Center for Neuroprosthetics, School of Life Sciences, Ecole Polytechnique Fédérale de Lausanne, Switzerland

^3^ Department of Neurology, Inselspital, Bern University Hospital, University of Bern, Switzerland

^4^ CORE Lab, Psychosomatic Competence Center, Department of Neurology, Inselspital, Bern University Hospital, University of Bern, Switzerland

^5^ Department of Neurology, University Hospital Geneva, Switzerland

^6^ University Neurorehabilitation, Department of Neurology, Inselspital, Bern University Hospital, University of Bern, Switzerland

^*^ Joint first authorship

**Methods**

**Neuropsychological examination**

Most of the patients underwent detailed neuropsychological examinations, including assessment of verbal and visuo-spatial long-term memory, attention, executive functions visuo-construction and visuo-spatial perception by means of standardised tests. Most commonly used was a battery with norms for the Swiss population [1] including tests of memory, executive functions and visuo-construction. In some patients, the CERAD [2] or the Hopkins Verbal Learning Test [3] were administered. Attention was commonly assessed by use of the computerised Test for Attentional Performance (TAP) [4]. Based on these assessments and on other clinical records and reports, performance levels were collated and scored for each patient and domain separately (see Supplementary Table 1).

Results of the neuropsychological evaluation were analyzed using a chi-square test for independent samples, or the Fisher’s exact test, respectively, if the expected frequencies were < 1. The p-value was adjusted for multiple comparisons using the Holmes-Bonferroni method.

**Lesion analysis**

All imaging data was reviewed for the purpose of this study by two of the authors (LH and AD) in order to determine the lesion location. For lesion overlap and statistical analysis, we used MRIcron and Non Parametric Mapping (NPM), which is part of the MRIcron software package [5]. Anatomical structures were labelled according to the AAL atlas implemented in MRIcron (http://www.mccauslandcenter.sc.edu/mricro/mricron).

In order to illustrate the neural correlates underlying topographical disorientation (TD), we subsequently traced the lesion for each patient on the T1 template using MRIcron. Structural lesions were identified by MRI or CT. MRI brain scans were normalized to the smoothed T1 template using SPM5 (http://www.fil.ion.ucl.ac.uk/spm/software/spm5)[6]. Since unified segmentation models give the most precise registration of lesioned structural images [7], no cost-function masking was necessary. If only a CT scan was available, lesions were traced manually slice by slice on the T1 weighted images using MRIcron. The later manual tracing on the template brain was only done when confidence could be achieved for matching corresponding slices between the lesioned brain and the template brain. No patients with unclear lesion boundaries or metallic artefacts were included in the analysis. Lesion volumes (volume of interest, VOI) were determined as the sum of all voxels compromising the traced lesion in all slices and were spatially smoothed using a 3mm full width at half maximum (FWHM) Gaussian Kernel and a threshold of 0.5. The same procedure was applied to the control group.

**Lesion network mapping analysis**

In addition to the lesion analysis, we applied lesion network mapping analysis, a method developed by Boes and colleagues [8] that enabled us to investigate the network associated with TD without the need of functional imaging data from patients. This method is very useful when the symptom is thought to arise from a network rather than from a single region [9]. To this aim, the TD lesions were used as seed region of interest (ROI) in a resting state analysis with data from 126 healthy subjects of the Enhanced Nathan Kline Institute Rockland Sample [10] as described in Blondiaux et al. [11]

The lesions masks were used as seed ROIs, and their mean time course was extracted and correlated to all other brain voxels using the Functional Connectivity (CONN) toolbox (v.18.a, http://www.nitrc.org/projects/conn)[12]. The whole brain analysis was restricted to the grey matter voxels. Each remaining lesion-seed yielded a brain network thresholded at t > ± 4.25 with p < 0.00005 peak-level uncorrected [8,13]. The networks were then binarized and overlapped to determine the regions of shared positive and negative correlations. The network overlap was thresholded at 90 % (at least 9 cases out of 10) with a minimal cluster extent of 10 voxels. This procedure was repeated with the lesions of the control group.

Next, in order to determine the regions specific to TD, we compared the TD-derived network with the control-derived network using VLSM and the Liebermeister test. The analysis was performed within the 90% RP- network overlap in order to assess its specificity compared to the control group with spatial disorientation. Only voxels showing 30% of overlap across the TD patients were considered and voxels were considered significant according to FWE correction (with 4000 permutations, only clusters larger than 10 voxels and in the grey matter are reported). Since the Liebermeister test is a binary test indicating whether or not the voxel is connected to the network, the positive and negative maps were analyzed separately.

**The study analyses were not pre-registered prior to the research being conducted.**

**References**

1 Balzer C, Berger JM, Caprez G, *et al.* *Materialien und Normwerte fuer die neuropsychologische Diagnostik (MNND)*. Rheinfelden, Switzerland: : Normdaten 2011.

2 Aebi C, Bern R. *Validierung der neuropsychologischen Testbatterie Eine Multi-Center Studie*. Psychiatrie. 2002.

3 Benedict RHB, Schretlen D, Groninger L, *et al.* Hopkins Verbal Learning Test – Revised: Normative Data and Analysis of Inter-Form and Test-Retest Reliability. *https://doi.org/101076/clin121431726* 2010;**12**:43–55. doi:10.1076/CLIN.12.1.43.1726

4 Zimmermann P, Fimm B. *Test for attentional performance (TAP)*. Herzogenrath, Germany: : PsyTest 1995.

5 Rorden C, Karnath H-O, Bonilha L. Improving lesion-symptom mapping. *J Cogn Neurosci* 2007;**19**:1081–8. doi:10.1162/jocn.2007.19.7.1081

6 Ashburner J, Friston KJ. Unified segmentation. *Neuroimage* 2005;**26**:839–51. doi:S1053-8119(05)00110-2 [pii]10.1016/j.neuroimage.2005.02.018

7 Crinion J, Ashburner J, Leff A, *et al.* Spatial normalization of lesioned brains: performance evaluation and impact on fMRI analyses. *Neuroimage* 2007;**37**:866–75. doi:10.1016/j.neuroimage.2007.04.065

8 Boes AD, Prasad S, Liu H, *et al.* Network localization of neurological symptoms from focal brain lesions. *Brain* Published Online First: 2015. doi:10.1093/brain/awv228

9 Fox MD. Mapping symptoms to brain networks with the human connectome. *N Engl J Med* Published Online First: 2018. doi:10.1056/NEJMra1706158

10 Nooner KB, Colcombe SJ, Tobe RH, *et al.* The NKI-Rockland Sample: A Model for Accelerating the Pace of Discovery Science in Psychiatry. *Front Neurosci* 2012;**6**:1–11. doi:10.3389/fnins.2012.00152

11 Blondiaux E, Heydrich L, Blanke O. Common and distinct brain networks of autoscopic phenomena. *NeuroImage Clin* 2021;:102612. doi:10.1016/j.nicl.2021.102612

12 Whitfield-Gabrieli S, Nieto-Castanon A. Conn: A Functional Connectivity Toolbox for Correlated and Anticorrelated Brain Networks. *Brain Connect* Published Online First: 2012. doi:10.1089/brain.2012.0073

13 Darby RR, Laganiere S, Pascual-Leone A, *et al.* Finding the imposter: Brain connectivity of lesions causing delusional misidentifications. *Brain* 2017;**140**:497–507. doi:10.1093/brain/aww288
