# Supplementary material for: The neural correlates of topographical disorientation—a lesion analysis study
Source: Ann Clin Transl Neurol. 2024 Jan 17;11(2):520–4. doi: 10.1002/acn3.51967 (PMC10863913; doi:10.1002/acn3.51967)
Supplement: Supplementary file 2 — Table S1. Neuropsychological profile of patients with TD and the control group. [file ACN3-11-520-s003.docx]

|  | **Hand** | **Lesion**  **Type** | **Lesion Side** | **Confabulations** | **Memory**  **verbal** | **Memory**  **visuo-spatial** | **Attention** | **Executive functions** | **Visuo-construction** | **Visual perception/ visual field** | **Neglect** | **Language** | **Sensorimotor** |  |
| --- | --- | --- | --- | --- | --- | --- | --- | --- | --- | --- | --- | --- | --- | --- |
| **CG** | r | Tumour | right | - | ++ | + | - | +++ | - | - | - | - | - |  |
| **CG** | r | TBI | right | - | ++ | +++ | +++ | +++ | ++ | - | - | - | - |  |
| **CG** | r | Stroke (isch.) | right | - | n/a | n/a | n/a | n/a | n/a | left | - | - | - |  |
| **CG** | r | Stroke (isch.) | bilateral | - | ++ | ++ | n/a | +++ | n/a | n/a | - | - | - |  |
| **CG** | r | Stroke (RA) | right | + | +++ | n/a | +++ | +++ | n/a | hall. | left | - | + |  |
| **CG** | r | Stroke (isch.) | right | - | ++ | n/a | +++ | +++ | +++ | left | left | - | + |  |
| **CG** | r | TBI | right | + | +++ | +++ | +++ | +++ | - | - | - | + | + |  |
| **TD** | r | Stroke | right | - | + | +++ | ++ | + | ++ | left | left | - | + |  |
| **TD** | r | Stroke (isch.) | right | - | - | ++ | - | - | - | left (UQ) | - | - | - |  |
| **TD** | r | Stroke (isch.) | bilateral | - | - | +++ | +++ | - | + | left | left | - | - |  |
| **TD** | r | Stroke (isch.) | bilateral | + (prov) | +++ | - | ++ | ++ | ++ | - | - | ++ | + |  |
| **TD** | r | Encephalitis | right | - | + | +++ | ++ | + | ++ | left (LQ) | - | - | - |  |
| **TD** | r | Stroke (isch.) | right | - | + | +++ | ++ | ++ | +++ | left | left | - | + |  |
| **TD** | r | Stroke (isch.) | right | - | - | +++ | - | - | - | left (UQ) | - | - | - |  |
| Scoring: - = no impairment described, + = mild impairment (up to 1.5 standard deviations (SD) below norm), ++ = moderate impairment (between 1.5 and 2.5 SD below norm), +++ = severe impairment (more than 2.5 SD below norm), n/a = no description available.  Abbreviations: hall. = hallucinations, isch. = ischemic, LQ/UQ = lower/upper quadrant, prov. = provoked, RA = ruptured aneurysm, CG = control group, TD = topographical disorientation, TBI = traumatic brain injury. | | | | | | | | | | | | | | |
