# Supplementary material for: The neural correlates of topographical disorientation—a lesion analysis study
Source: Ann Clin Transl Neurol. 2024 Jan 17;11(2):520–4. doi: 10.1002/acn3.51967 (PMC10863913; doi:10.1002/acn3.51967)
Supplement: Supplementary file 3 — Table S2. Brain regions functionally connected to all lesion locations in TD. [file ACN3-11-520-s001.docx]

| Brain regions | Voxel size | Intensity | x | y | z |
| --- | --- | --- | --- | --- | --- |
| ***Positive correlations*** |  |  |  |  |  |
| R. lingual gyrus /calcarine gyrus | 1521 | 2.70 | 20 | -95 | -12 |
| L. lingual gyrus/precuneus/calcarine gyrus | 558 | 2.70 | -9 | -48 | 2 |
|  | 12 | 2.70 | -21 | -95 | -15 |
| R. cerebellum | 862 | 2.70 | 33 | -38 | -27 |
|  | 329 | 3.19 | 5 | -78 | -42 |
|  | 59 | 3.19 | 11 | -51 | -44 |
|  | 41 | 2.7 | 14 | -66 | -26 |
|  | 63 | 2.23 | 32 | -65 | -54 |
| L. Cerebellum | 168 | 3.19 | -6 | -62 | -47 |
|  | 71 | 2.70 | -24 | -87 | -21 |
|  | 45 | 2.70 | -20 | -44 | -47 |
|  | 12 | 2.23 | -33 | -58 | -21 |
| L. fusiform gyrus | 284 | 2.70 | -20 | -35 | -18 |
| R. hippocampus | 22 | 2.23 | 33 | -30 | -5 |
| L. hippocampus | 40 | 2.70 | -18 | -33 | -2 |
| R. thalamus | 12 | 2.70 | 17 | -32 | 9 |
